# The Effectiveness of Ketorolac in Relieving Pain Associated With Root Canal Therapy: A Systematic Review and Meta‐Analysis

**DOI:** 10.1002/cre2.70295

**Published:** 2026-02-17

**Authors:** Ruijing Ping, Xiaoning Kang, Ruizhen Fang, Haozhen Wang, Li‐an Wu

**Affiliations:** ^1^ State Key Laboratory of Oral & Maxillofacial Reconstruction and Regeneration, National Clinical Research Center for Oral Diseases, Shaanxi Clinical Research Center for Oral Diseases, Department of Pediatric Dentistry, School of Stomatology The Fourth Military Medical University Xi'an China

**Keywords:** analgesic effects, endodontics, ketorolac, pain evaluation, pulpitis

## Abstract

**Objectives:**

Effective management of post‐endodontic pain is crucial for patient comfort. Although ketorolac is used for this purpose, its efficacy relative to other analgesics remains uncertain. This study systematically assesses evidence from randomized clinical trials that investigate the comparative effectiveness of ketorolac and other analgesic agents for the control of post‐endodontic pain.

**Material and Methods:**

This study conducted a comprehensive search across several databases, including PubMed, Web of Science, ScienceDirect, Cochrane Library, Embase, Scopus, SinoMed, CNKI, Webvpn, and Wanfang Data. Search deadline was around August 2025. The study employed the inverse variance approach and a random effects model to analyze continuous data, utilizing the standardized mean as the comprehensive effect indicator. For dichotomous data, this study applied the risk ratio (RR), fixed‐effect analysis model, and Mantel–Haenszel statistical methods.

**Results:**

Our database search identified 386 potentially relevant studies. Ultimately, seven studies were selected for qualitative analysis, and five were chosen for quantitative analysis. The qualitative assessment indicated that five studies favored ketorolac, one study reported similar analgesic effects between ketorolac and alternative medications, while one study concluded that ketorolac was less effective. The quantitative analysis demonstrated a statistically significant difference in the analgesic effect of ketorolac compared to other drugs at 6 h post‐administration. However, the analgesic effects of ketorolac did not show statistically significant differences compared to other medications at 12 or 24 h post‐administration. Additionally, a lower proportion of patients required supplementary drug treatment after receiving ketorolac compared to those treated with other analgesics.

**Conclusion:**

This meta‐analysis concluded that ketorolac is effective for managing post‐endodontic pain within the first 6 h and reduces the need for rescue medication compared to alternatives, thereby supporting its use as a favorable option following root canal treatment.

## 1 Background

Root canal treatment (RCT) is the primary intervention for symptomatic pulpitis, aimed at the complete eradication of the infectious source and the resolution of acute symptoms (Kohli et al. 2021). As an invasive procedure, however, it inevitably induces iatrogenic inflammation in the periapical tissues, consequently leading to postoperative pain. This makes adjunctive analgesia a critical component of post‐RCT management (Kumar et al. 2025; Santos‐Puerta and Peñacoba‐Puente 2022). Non‐steroidal anti‐inflammatory drugs (NSAIDs), a class of heterogeneous acidic compounds, are widely utilized for post‐endodontic pain control due to their antipyretic, analgesic, and anti‐inflammatory properties (Kotowska‐Rodziewicz et al. 2023; Terzi et al. 2018). A single oral dose of Naproxen and Novafen (which contains paracetamol, ibuprofen, and caffeine anhydrous) administered immediately after treatment has been shown to be more effective than oral Tramadol in relieving pain following pulpectomy and root canal preparation (Mehrvarzfar et al. 2012). Patients experiencing symptomatic irreversible pulpitis may experience pain relief for up to 24 h by taking dexamethasone orally or by injecting it primarily intraligamentarily and supraperiosteally into the root canal (Nogueira et al. 2018). A recent survey indicated that ketorolac, as an NSAID, is among the preferred prescriptions for comprehensive dental pain management due to its rapid onset of pain relief and overall effectiveness (Shukla et al. 2024).

Ketorolac is an NSAID known for its potent analgesic properties, alongside moderate anti‐inflammatory and antipyretic effects. In standard analgesic animal models, ketorolac demonstrates an analgesic activity that is 800 times greater than that of aspirin, surpassing the efficacy of indomethacin and naproxen, and is comparable to or exceeds that of prednisone. Clinical studies have demonstrated that ketorolac can alleviate moderate to severe pain in patients with conditions such as renal colic, migraine, musculoskeletal pain, or sickle cell crisis, showing efficacy equivalent to commonly used opioids like morphine and meperidine (Gillis and Brogden 1997). A significant advantage of ketorolac over opioids is its lack of adverse effects on respiration and its non‐association with central nervous system side effects, abuse, or addictive potential, rendering it a safer option for long‐term pain management. The most commonly reported adverse effects of ketorolac include dizziness, nausea, and vomiting, which are typically mild and manageable for patients (Litvak and McEvoy 1990). Ketorolac can be administered orally via tromethamine or through periapical, intravenous (IV), or intramuscular (IM) injection. Notably, it is the only NSAID that can be delivered via the nasal route, with Sprix® being an intranasal formulation of ketorolac (Snyder and Bregmen 2012). Additionally, Acular®, another formulation of ketorolac, is utilized to treat ocular conditions by providing anti‐inflammatory and analgesic effects (Vadivelu et al. 2015).

Current meta‐analyses support the analgesic efficacy of ketorolac in oral surgery. Specifically, postoperative administration of 30 mg ketorolac has been demonstrated to significantly reduce pain following third molar extraction, while also exhibiting a more favorable side effect profile compared to alternative analgesics (Isiordia‐Espinoza et al. 2022). Furthermore, the efficacy of a standard alveolar nerve block for irreversible pulpitis may be enhanced by the pre‐treatment with ketorolac prior to root canal therapy (Sivaramakrishnan and Sridharan 2018). Although ketorolac is frequently employed in the management of post‐endodontic pain, there is a scarcity of evidence from systematic reviews and meta‐analyses on its efficacy. Consequently, this study is designed to perform a meta‐analysis to systematically evaluate the effectiveness of ketorolac compared to other analgesic drugs in alleviating pain associated with root canal treatment.

## 2 Materials and Methods

### 2.1 Protocol and Registration

This meta‐analysis followed the Preferred Reporting Items for Systematic Reviews and Meta‐Analyses (PRISMA) guidelines and has been pre‐registered in the PROSPERO database with registration number CRD42024535882.

### 2.2 Search Strategy

The studies included in this review can be found in the following databases: PubMed, Web of Science, ScienceDirect, Cochrane Library, Embase, Scopus, SinoMed, CNKI databases, Webvpn databases, and Wanfang data. Search deadline was around August 2025. The search terms used were (Ketorolac OR Ketorolac Tromethamine OR Acular OR Toradol) AND (Pulpitis OR Pulpitides OR Endodontic Inflammation OR Endodontic Inflammations) AND (Pain OR Ache OR Toothache OR Odontalgia OR Acute Pain OR Acute Pains OR Chronic Pain OR Toothaches OR Chronic Primary Pain OR Odontalgias OR Chronic Secondary Pain OR Widespread Chronic Pain). Each database had its search format, so the detailed search methods were slightly different. Supporting materials describe the detailed search methods used by each database (Supporting Information: S1).

### 2.3 Inclusion and Exclusion Criteria

This meta‐analysis developed the inclusion criteria for this study based on the PICOS (Population, Intervention, Comparison, Outcome, and Study Design) framework. Below was a description of each domain:

The inclusion criteria were:

1. Population (P): Patients diagnosed with irreversible pulpitis, defined according to AAE guidelines as a clinical condition for teeth characterized by persistent spontaneous pain or lingering pain in response to thermal stimuli (especially cold), lasting for seconds to minutes after stimulus' removal. These teeth had vital pulp responses to electric or cold testing, were structurally sound, and showed no periapical pathology on radiographs.
2. Intervention (I): The perioperative administration of ketorolac during root canal treatment, aimed at reducing postoperative endodontic pain.
3. Comparison (C): Compared with patients who use other medications to alleviate pain.
4. Outcome (O): Use VAS or Heft–Parker to measure initial pain intensity and pain intensity at different periods after administration, the number of patients who needed further medication treatment after taking ketorolac.
5. Study design (S): Randomized clinical trials.

The exclusion criteria were:

1. Reviews or commentaries.
2. Only ketorolac was used in the study without comparison with other drugs.
3. Unable to obtain full text or missing data.

### 2.4 Evaluation of Risk of Bias

Two researchers independently conducted literature searches, data extraction, and quality assessments. Any discrepancies were resolved by consensus through discussion with a third researcher. The extracted data encompassed the study ID, patient characteristics, trial grouping, pain assessment methods, administration methods, pain assessment timings, conclusions, the number of participants requiring additional medication, and reported side effects. The risk of bias in the included studies was assessed using the Cochrane Handbook 5.1.0, which outlines seven domains: random sequence generation, allocation concealment, blinding of participants and personnel, blinding of outcome assessment, incomplete outcome data, selective outcome reporting, and other potential sources of bias. Each entry was evaluated with a designation of “yes,” indicating a low probability of bias; “no,” reflecting a high risk of bias; or “unclear,” denoting either insufficient data or uncertainty regarding the potential for bias.

### 2.5 Statistical Analysis

We utilized RevMan 5.3 to analyze the extracted data. This study employed the inverse variance method and a random effects model to assess continuous data, using the standardized mean difference (SMD) as the overall effect indicator. For dichotomous data, we applied the risk ratio (RR), a fixed‐effect analysis model, and the Mantel–Haenszel statistical methods. To evaluate heterogeneity, we used the I^²^ statistic and the Cochran Q test. All analyses were conducted at a significance level of 95%.

## 3 Results

### 3.1 Study Selection

**Figure 1:**
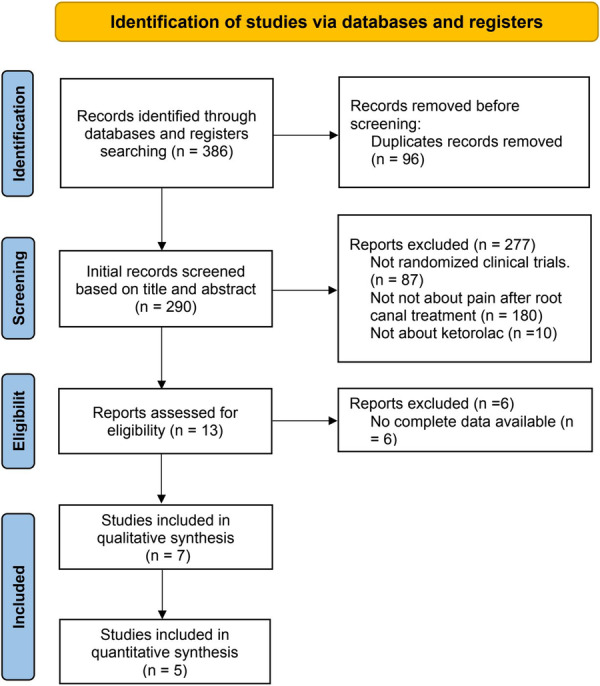
Study flow diagram.

The outline of the search is presented in Figure 1. A search of the database identified 386 potentially related studies. After removing duplicates, 290 studies were retained. Upon reviewing the titles and abstracts, 13 studies remained. We then examined the full texts of these 13 articles and found that six studies lacked complete data. Consequently, seven studies were included for qualitative analysis (Akhlaghi et al. 2019; Penniston and Hargreaves 1996; Praveen et al. 2017; Rogers et al. 1999; Sekhar et al. 2024; Sethi et al. 2014; Turner et al. 2011). Of these, two studies were unable to provide extractable data, leading to a quantitative analysis based on the remaining five investigations (Akhlaghi et al. 2019; Penniston and Hargreaves 1996; Praveen et al. 2017; Rogers et al. 1999; Sethi et al. 2014).

### 3.2 Characteristics of the Included Articles

Table 1 presents detailed information on the seven selected studies, which were published between 1994 and 2024. The included studies exhibited heterogeneity in both the administration protocols and the pain assessment scales employed. Regarding the administration protocol, among the seven studies, five implemented a pre‐operative regimen, one applied ketorolac as an intracanal medicament during the procedure, and one adopted a postoperative protocol. In terms of specific routes of administration, three studies utilized the oral route, three employed local injection, and one adopted an intranasal approach. For pain assessment, the following instruments were used across the studies: the Heft–Parker scale, Verbal Rating Scale, 0–10 Visual Analog Scale (VAS), 0–100 VAS, and a 0–170 VAS.

**Table 1:** Characteristics of the included studies.

Study ID	Details of patients	Groups/drug	Pain evaluation	Administration method	Time of evaluation of pain	Conclusion	No. of patients requiring additional medications	Reported side effects
Penniston and Hargreaves (1996)	Patient selects root canal therapy for pain originating from a vital/nonvital tooth. The pain on the Heft–Parker pain scale should be at least 30 mm (0–160 scale).	Group A: intramuscular placebo and periapical placebo (*n* = 14) Group B: 30 mg intramuscular ketorolac and periapical placebo (*n* = 10) Group C: 30 mg intramuscular placebo and periapical ketorolac (*n* = 18) Group D: intramuscular placebo and periapical local anesthesia (2% mepivacaine with 1:20,000 levonordefrin) (*n* = 10)	VAS 0–100 and Heft‐Parker	Intramuscular injection and periapical infiltration injection	Following the drug injections, pain scores were recorded at 15, 30, 45, and 60 min, and record pain at 3, 4, 5, and 6 h after pulpotomy is completed.	Intraoral injection of ketorolac may prove to be a useful adjunct in the management of endodontic pain patients. Further studies are required to replicate these findings and to develop optimal treatment combinations.	Group A: 10 Group B: 2 Group C: 6 Group D: 5	NR
Rogers et al. (1999)	The patient was diagnosed with an irreversible pulpitis or normal, but in need of endodontic therapy.	Group A: 600 mg oral ibuprofen (*n* = 12) Group B: placebo (*n* = 12) Group C: 4 mg/mL dexamethasone (*n* = 12) Group D: 60 mg/2 ml ketorolac tromethamine (n = 12)	VAS 0–100	Intracanal medication	After initiation of root canal treatment, pain scores were recorded at 6, 12, 24, and 48 h.	No significant differences were demonstrated between ibuprofen and either dexamethasone or ketorolac.	Group A: 3 Group B: 6 Group C: 2 Group D: 1	NR
Turner et al. (2011)	The patient reported a verbal numerical score of 3 or greater (out of 10) for pain and had symptoms of irreversible pulpitis or pulp necrosis, or had previously received a root diagnosis of normal, acute periapical periodontitis, acute periapical abscess, chronic periapical periodontitis, or chronic periapical abscess.	Group A: 30 mg/mL ketorolac (*n* = 10) Group B: placebo (*n* = 11)	Categoric, VAS 0–100 and Heft‐Parker 0–170	Intranasal delivery	Pain levels were recorded at 15 and 30 min after the initial IN dosing (before endodontic treatment); 30 min after completion of endodontic treatment; and 4, 8, and 12 h after the initial IN spray.	These results suggest that IN ketorolac may provide a novel and efficacious method for pain relief in endodontic pain patients.	NR	NR
Sethi et al. (2014)	The patient was diagnosed with symptomatic irreversible pulpitis and baseline pain scores greater than 3 cm on visual analog scale.	Group A: 100 mg tapentadol (*n* = 18) Group B: 400 mg etodolac (*n* = 19) Group C: 10 mg ketorolac (*n* = 19)	VAS 0–10	Oral medication	Patients completed the questionnaire at 0, 6, 12, 18, and 24 h after completion of root canal treatment.	Single oral dose of 10 mg of ketorolac and 100 mg of tapentadol as a pretreatment analgesic significantly reduced postoperative endodontic pain in patients with symptomatic irreversible pulpitis when compared to 400 mg of etodolac.	NR	Mild headache and mild dizziness.
Praveen et al. (2017)	The patient was diagnosed with a pulpal necrosis or irreversible pulpitis.	Group A: 20 mg ketorolac (*n* = 29) Group B: 30 mg prednisolone (*n* = 30) Group C: placebo (*n* = 27)	VAS 0–10	Oral medication	After root canal treatment, pain scores were recorded at 6, 12, 24, and 48 h.	From this study, it could be concluded that a single pretreatment dose of prednisolone has a more sustained effect in reducing postendodontic pain compared with placebo or ketorolac.	NR	NR
Akhlaghi et al. (2019)	The patient was diagnosed with irreversible pulpitis and the Heft–Parker Visual Analog Scale [HP‐VAS] was greater than 54.	Group A: 30 mg/mL ketorolac tromethamine (*n* = 30) Group B: normal saline (*n* = 30)	Heft–Parker	Periapical infiltration injection	After root canal treatment completion, pain scores were recorded at 2, 4, 6, and 24 h.	Ketorolac buccal infiltration could reduce the postoperative pain experienced by patients requiring endodontic treatment diagnosed with symptomatic irreversible pulpitis.	Group A: 12 Group B: 17	NR
Sekhar et al. (2024)	The patients were diagnosed with symptomatic irreversible pulpitis.	Group A: 2 mL sterile saline (*n* = 30) Group B: 4 mg/mL dexamethasone (*n* = 30) Group C: 30 mg/mL ketorolac tromethamine (*n* = 30)	VRS	Intramuscular injection	Postoperatively, the incidence and severity of pain were recorded after four, 24, and 48 h were recorded after four, 24, and 48 h.	Effective and complete debridement of infected root canal system provides predictable gradual reduction of post‐endodontic pain.	NR	NR

Abbreviations: ID, identification; IN, intranasal; NR, not reported; SD, standard deviation; VAS, visual analog scale; VRS, verbal rating scale.

Among the seven studies, three utilized additional medications, suggesting that the incidence of using supplementary drugs following ketorolac administration was lower compared to other medications (Akhlaghi et al. 2019; Penniston and Hargreaves 1996; Rogers et al. 1999). Concerning side effects, only one study reported this information (Sethi et al. 2014). The most commonly noted adverse effects of ketorolac included mild headache, dizziness, sweating, and pain at the injection site.

### 3.3 Assessing Risk of Bias

**Figure 2:**
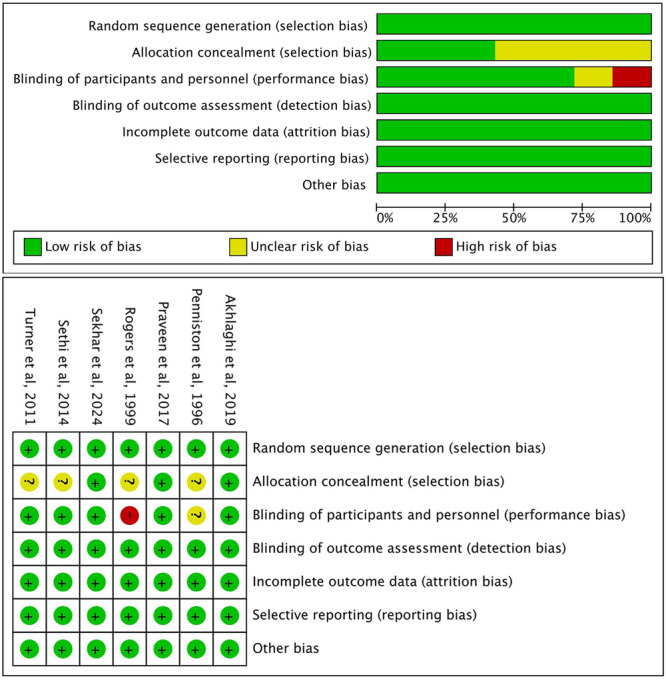
Risk of bias assessment.

The risk of bias for the selected studies is illustrated in Figure 2. Three studies were classified as having a low risk of bias (Akhlaghi et al. 2019; Praveen et al. 2017; Sekhar et al. 2024). Allocation concealment was deemed unclear in four studies (Penniston and Hargreaves 1996; Rogers et al. 1999; Sethi et al. 2014; Turner et al. 2011). The blinding of participants and operators was also unclear in one study (Penniston and Hargreaves 1996). Additionally, one study was assessed as high risk for blinding of participants and operators due to the use of different administration methods (Rogers et al. 1999).

## 4 Meta‐Analysis

### 4.1 Qualitative Analysis

A qualitative evaluation was conducted using seven reports, of which five demonstrated favorable results for ketorolac (Akhlaghi et al. 2019; Penniston and Hargreaves 1996; Sekhar et al. 2024; Sethi et al. 2014; Turner et al. 2011). The findings from one clinical trial indicated no significant differences in the analgesic effects among ketorolac, dexamethasone, or ibuprofen (Rogers et al. 1999). Additionally, one clinical trial found that prednisolone reduces postendodontic pain more effectively over time (Praveen et al. 2017).

### 4.2 Quantitative Analysis

**Figure 3:**
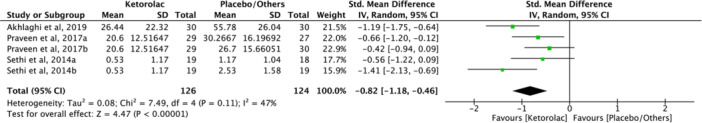
Forest plot comparing the pain risk of patients in the ketorolac group and other drug groups at 6 h after root canal therapy. CI, confidence interval; SD, standard deviation.

Among the seven included studies, three studies evaluated the risk of pain at 6, 12, and 24 h after root canal therapy, with complete data available (Akhlaghi et al. 2019; Praveen et al. 2017; Sethi et al. 2014). Therefore, based on the data from three clinical studies, the risk of pain at 6 h after root canal therapy was evaluated. The outcomes of the meta‐analysis demonstrated statistical differences between ketorolac and other medications (SMD = −0.82, *I* ^2^ = 47%, *Z* = 4.47, 95% CI = −1.18 to −0.46, *p* < 0.00001, Figure 3).

**Figure 4:**
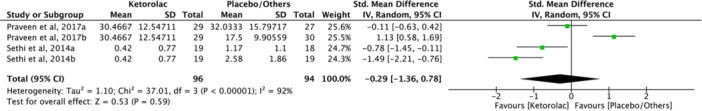
Forest plot comparing the pain risk of patients in the ketorolac group and other drug groups at 12 h after root canal therapy. CI, confidence interval; SD, standard deviation.

According to data from two clinical studies evaluating pain for 12 h, (Praveen et al. 2017; Sethi et al. 2014) the meta‐analytical evaluation showed no statistically significant difference between ketorolac and other analgesics (SMD = −0.29, *I* ^2^ = 92%, *Z* = 0.53, 95% CI = −1.36 to 0.78, *p* = 0.59, Figure 4).

**Figure 5:**
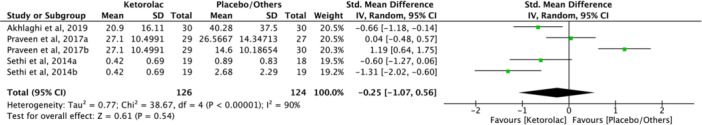
Forest plot comparing the pain risk of patients in the ketorolac group and other drug groups at 24 h after medication. CI, confidence interval; SD, standard deviation.

According to three studies evaluating 24‐h pain, (Akhlaghi et al. 2019; Praveen et al. 2017; Sethi et al. 2014) there was no statistically significant difference between ketorolac and other medications (SMD = −0.25, *I* ^2^ = 90%, *Z* = 0.61, 95% CI = −1.07 to 0.56, *p* = 0.54, Figure 5).

**Figure 6:**
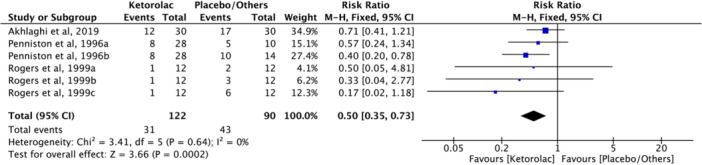
Forest plot comparing the number of patients using additional drugs between the ketorolac group and other drug groups. CI, confidence interval.

The overall evaluation of the number of patients who require additional medication included three clinical trials (Akhlaghi et al. 2019; Penniston and Hargreaves 1996; Rogers et al. 1999). Meta‐analysis evaluation showed statistical differences between ketorolac and other drugs (RR = 0.50, *I* ^2^ = 0%, *Z* = 3.66, 95% CI = 0.35–0.73, *p* = 0.0002, Figure 6). Compared to those who take other medications, fewer patients needed extra medication after taking ketorolac.

## 5 Discussion

This meta‐analysis demonstrates that ketorolac is an effective analgesic for managing postendodontic pain, exhibiting significant superiority over several common comparators, including dexamethasone, ibuprofen, tapentadol, etodolac, and prednisolone, particularly in the early postoperative period. The included studies, despite their heterogeneity in administration routes (oral, intranasal, and local injection), consistently support ketorolac's potent analgesic profile. For instance, oral ketorolac was shown to outperform other oral agents (Praveen et al. 2017; Rogers et al. 1999; Sethi et al. 2014), while local delivery methods, including intracanal placement and periapical injection, also yielded significant pain relief (Akhlaghi et al. 2019; Penniston and Hargreaves 1996; Sekhar et al. 2024). The efficacy of a novel intranasal (IN) formulation was also established, highlighting its potential as a noninvasive alternative (Turner et al. 2011).

The quantitative results of this study indicated that ketorolac demonstrated significant analgesic effects when compared to dexamethasone, ibuprofen, tapentadol, etodolac, and prednisolone at 6 h post‐administration. This observation may be attributed to the pharmacological mechanisms and pharmacokinetics of the drug. Ketorolac primarily functions by inhibiting the cyclooxygenase enzyme involved in arachidonic acid metabolism, thereby reducing prostaglandin synthesis (Fan et al. 2025; Redden 1992). Ketorolac demonstrates favorable pharmacokinetic characteristics, including rapid absorption and onset of action. After intramuscular or intravenous administration, analgesia typically begins within approximately 10 min, peaking between 75 and 150 min. Intranasal administration further shortens the time to peak concentration to about 45 min, while oral administration achieves onset within 30–60 min, with peak plasma concentrations occurring in 1.5–4 h (Brocks and Jamali 1992). In comparison, the comparator drugs generally exhibit longer times to peak effect: approximately 60 min for dexamethasone, around 80 min for etodolac, 1.5–3 h for ibuprofen, (Cajaraville 2021) about 2 h for prednisolone, and an even more delayed peak for tapentadol (Deeks 2018). These differences in absorption and time to peak concentration may account for ketorolac's superior analgesic performance in the early postoperative period. However, at the 12‐h mark, no significant difference in analgesic effects was observed between ketorolac and the other drugs, likely due to its plasma half‐life of approximately 6 h, after which inflammation may exacerbate, resulting in a decline in ketorolac's analgesic efficacy.

Pain is a prevalent symptom encountered in the emergency department. Oral analgesics are often influenced by gastrointestinal conditions, which may lead to reduced bioavailability and diminished therapeutic efficacy (Guo and Pratap Singh 2019). Although intramuscular injection can directly target inflammatory sites, its onset of action may be delayed, and discomfort at the injection site can easily provoke patient apprehension (Ayinde et al. 2021). In contrast, IN drug delivery capitalizes on the extensive surface area of mucosal tissue in the nasal cavity and sinuses, facilitating rapid absorption into the bloodstream and quicker onset of action (Falatah et al. 2023; Zanza et al. 2023). Few analgesics have been evaluated for IN administration, with ketorolac being the sole NSAID approved for nasal delivery. Among the analgesics suitable for intranasal delivery, ketorolac is currently the only nonsteroidal anti‐inflammatory drug approved for this route (Kress et al. 2009; McAleer et al. 2007; Telfer et al. 2009). Studies have demonstrated that intranasal ketorolac spray provides superior analgesia compared to intravenous administration following mandibular fracture surgery, while exhibiting comparable efficacy to the intravenous formulation in treating pediatric migraine (Tsze et al. 2022). Furthermore, for moderate‐to‐severe acute pain in children (such as abdominal pain, trauma, and migraine), intranasal analgesia may serve as an effective alternative to intramuscular injection and could partially replace intravenous administration (Cozzi et al. 2024; Prescott et al. 2023). In recent years, intranasal ketorolac has gained popularity in oral surgery. Turner et al. found that IN ketorolac significantly reduced both preoperative and postoperative pain during pulp treatment (Turner et al. 2011). Another study examined the impact of IN ketorolac combined with nitrous oxide/oxygen therapy on the success rate of inferior alveolar nerve block (IANB) anesthesia in patients with symptomatic irreversible pulpitis. This research revealed that, compared to nitrous oxide/oxygen alone, intranasal ketorolac pre‐medication did not significantly enhance the success rate of IANB but offered a valuable alternative to traditional non‐anesthetic drug delivery (Stentz et al. 2018). In conclusion, the favorable pharmacokinetic profile, characterized by rapid onset, good tolerability, and a noninvasive route of administration, establishes intranasal ketorolac as a valuable complementary option in the management of diverse acute and postoperative pain states.

The seven articles included in this study demonstrate significant heterogeneity, which can be attributed to several factors. Firstly, the studies employed various measurement methods to assess pain, including the 0–10 Visual Analog Scale, 0–100 Visual Analog Scale, 0–170 Visual Analog Scale, Heft–Parker Scale, and Verbal Rating Scale. Secondly, the methods of administration differed, with two studies utilizing intramuscular injection, two employing oral administration, and one implementing intranasal delivery. Additionally, one study applied ketorolac as a root canal sealant, while another administered it via periapical injection.

Our research has certain imperfection. Firstly, despite a comprehensive review of the literature, this study includes only a limited number of randomized controlled trials. Variations in comparison groups and the inability of some studies to provide raw data restrict the inclusion of only a few studies in each meta‐analysis. Additionally, many of the studies have small sample sizes, which precludes the possibility of conducting subgroup analyses on dosing frequency and route. To achieve more reliable results in future research, we recommend utilizing larger sample sizes and conducting more rigorous randomized controlled trials.

## 6 Conclusion

This meta‐analysis demonstrates that ketorolac is effective in alleviating postendodontic treatment pain within 6 h when compared to placebo and other medications, with a reduced number of patients requiring additional analgesics. Consequently, ketorolac represents a favorable therapeutic option for managing post‐endodontic pain. Furthermore, we advocate for future research to conduct large‐sample, multicenter, randomized controlled trials that utilize standardized pain assessment criteria to further evaluate the therapeutic effects of ketorolac in this context.

## Author Contributions

All authors contributed to the design of this meta‐analysis. Ruijing Ping and Xiaoning Kang undertook data collection and analysis and wrote the first draft of the manuscript. Ruizhen Fang and Haozhen Wang put forward suggestions for revision of the manuscript. Li‐an Wu revised the manuscript content. The manuscript was revised several times, and all authors approved the final version.

## Conflicts of Interest

The authors declare no conflicts of interest.

## Supporting information

Supplementary Material.

## Data Availability

This manuscript is a systematic review and meta‐analysis. The data supporting the findings of this study were extracted from previously published studies, which are all cited in the reference list. Furthermore, the complete data extraction sheet is provided in Table 1, and the corresponding data analyses are presented in Figures 2, 3, 4, 5, 6.
