# Supplementary material for: The Effectiveness of Ketorolac in Relieving Pain Associated With Root Canal Therapy: A Systematic Review and Meta‐Analysis
Source: Clin Exp Dent Res. 2026 Feb 17;12(2):e70295. doi: 10.1002/cre2.70295 (PMC12914131; doi:10.1002/cre2.70295)
Supplement: Supplementary file 1 — Supplementary Material. [file CRE2-12-e70295-s001.docx]

## SUPPLEMENTARY MATERIAL

**Database information retrieval:**

English database：PubMed、Web of science、ScienceDirect、Cochrane Library、embase、scopus

1. PubMed

Search: (((("Ketorolac Tromethamine"[Mesh]) OR ("Ketorolac"[Mesh])) OR ((Acular[Title/Abstract]) OR (Toradol[Title/Abstract]))) AND (("Pulpitis"[Mesh]) OR (((((Pulpitides[Title/Abstract]) OR (Inflammation, Endodontic[Title/Abstract])) OR (Endodontic Inflammation[Title/Abstract])) OR (Endodontic Inflammations[Title/Abstract])) OR (Inflammations, Endodontic[Title/Abstract])))) AND ((("Pain"[Mesh] OR "Acute Pain"[Mesh] OR "Chronic Pain"[Mesh]) OR ("Toothache"[Mesh])) OR ((((((((Ache[Title/Abstract]) OR (Toothaches[Title/Abstract])) OR (Odontalgia[Title/Abstract])) OR (Odontalgias[Title/Abstract])) OR (Acute Pains[Title/Abstract])) OR (Chronic Primary Pain[Title/Abstract])) OR (Chronic Secondary Pain[Title/Abstract])) OR (Widespread Chronic Pain[Title/Abstract])))

Number of detected records：7

1. **Web of science**

#1: (((TS=(ketorolac)) OR TS=(Ketorolac Tromethamine)) OR TS=(Acular)) OR TS=(Toradol)

#2:(((((TS=(Pulpitis)) OR TS=(Pulpitides)) OR TS=(Inflammation, Endodontic)) OR TS=(Endodontic Inflammation)) OR TS=(Endodontic Inflammations)) OR TS=(Inflammations, Endodontic)

#3:(((((((((((TS=(pain)) OR TS=(Ache)) OR TS=(Toothache)) OR TS=(Toothaches)) OR TS=(Odontalgia)) OR TS=(Odontalgias)) OR TS=(Acute Pain)) OR TS=(Acute Pains)) OR TS=(Chronic Pain)) OR TS=(Chronic Primary Pain)) OR TS=(Chronic Secondary Pain)) OR TS=(Widespread Chronic Pain)

#4: #1 AND #2 AND #3

Number of detected records：54

1. **ScienceDirect**

(Ketorolac OR Acular OR Toradol) AND (Pulpitis OR Endodontic Inflammation) AND (Pain OR Ache OR Toothache OR Odontalgia)

Number of detected records：149

1. **Cochrane library**

#1：MeSH descriptor: [Ketorolac] explode all trees

#2：MeSH descriptor: [Ketorolac Tromethamine] explode all trees

#3：(Acular):ti,ab,kw OR (Toradol):ti,ab,kw

#4：#1 OR #2 OR #3

#5：MeSH descriptor: [Pulpitis] explode all trees

#6：(Pulpitides):ti,ab,kw OR (Inflammation, Endodontic):ti,ab,kw OR (Endodontic Inflammation):ti,ab,kw OR (Endodontic Inflammations):ti,ab,kw OR (Inflammations, Endodontic):ti,ab,kw

#7：#5 OR #6

#8：MeSH descriptor: [Pain] explode all trees

#9：MeSH descriptor: [Toothache] explode all trees

#10：MeSH descriptor: [Acute Pain] explode all trees

#11：MeSH descriptor: [Chronic Pain] explode all trees

#12：(Ache):ti,ab,kw

#13：(Toothaches):ti,ab,kw OR (Odontalgia):ti,ab,kw OR (Odontalgias):ti,ab,kw OR (Acute Pains):ti,ab,kw

#14：(Chronic Primary Pain):ti,ab,kw OR (Chronic Secondary Pain):ti,ab,kw OR (Widespread Chronic Pain):ti,ab,kw

#15：#8 OR #9 OR #10 OR #11 OR #12 OR #13 OR #14

#16：#4 AND #7 AND #15

Number of detected records：12

1. **embase**

#1：'ketorolac'/exp

#2：'ketorolac trometamol'/exp

#3：acular:ti,ab,kw OR toradol:ti,ab,kw

#4：#1 OR #2 OR #3

#5：'pulpitis'/exp

#6：pulpitides:ti,ab,kw OR 'inflammation, endodontic':ti,ab,kw OR 'endodontic inflammation':ti,ab,kw OR 'endodontic inflammations':ti,ab,kw OR 'inflammations, endodontic':ti,ab,kw

#7：#5 OR #6

#8：'pain'/exp

#9：'tooth pain'/exp

#10：'chronic pain'/exp

#11：ache:ti,ab,kw OR toothaches:ti,ab,kw OR odontalgia:ti,ab,kw OR odontalgias:ti,ab,kw OR 'acute pains':ti,ab,kw OR 'chronic primary pain':ti,ab,kw OR 'chronic secondary pain':ti,ab,kw OR 'widespread chronic pain':ti,ab,kw OR 'acute pain':ti,ab,kw

#12：#8 OR #9 OR #10 OR #11

#13：#4 AND #7 AND #12

Number of detected records：17

1. **Scopus**

( ( TITLE-ABS-KEY ( ketorolac ) OR TITLE-ABS-KEY ( "ketorolac trometamol" ) OR TITLE-ABS-KEY ( acular ) OR TITLE-ABS-KEY ( toradol ) ) ) AND ( ( TITLE-ABS-KEY ( pulpitis ) OR TITLE-ABS-KEY ( pulpitides ) OR TITLE-ABS-KEY ( "Inflammation, Endodontic" ) OR TITLE-ABS-KEY ( "Endodontic Inflammation" ) OR TITLE-ABS-KEY ( "Endodontic Inflammations" ) OR TITLE-ABS-KEY ( "Inflammations, Endodontic" ) ) ) AND ( ( TITLE-ABS-KEY ( pain ) OR TITLE-ABS-KEY ( ache ) OR TITLE-ABS-KEY ( toothache ) OR TITLE-ABS-KEY ( toothaches ) OR TITLE-ABS-KEY ( odontalgia ) OR TITLE-ABS-KEY ( odontalgias ) OR TITLE-ABS-KEY ( "Acute Pain" ) OR TITLE-ABS-KEY ( "Chronic Pain" ) OR TITLE-ABS-KEY ( "Acute Pains" ) OR TITLE-ABS-KEY ( "Chronic Primary Pain" ) OR TITLE-ABS-KEY ( "Chronic Secondary Pain" ) OR TITLE-ABS-KEY ( "Widespread Chronic Pain" ) ) )

Number of detected records：38

Chinese database：webvpn、SinoMed、WanFang、CNKI

1. **webvpn：**

Retrieval type：(题名或关键词=(酮咯酸 OR 酮咯酸氨丁三醇)) AND (题名或关键词=(牙髓炎 OR 牙髓炎症)) AND(题名或关键词=(疼痛 OR 痛证 OR 牙痛 OR 牙疼痛))

Number of detected records：0

1. **SinoMed：**

Retrieval type：("疼痛"[常用字段:智能] OR "痛证"[常用字段:智能] OR "牙痛"[常用字段:智能] OR "牙疼痛"[常用字段:智能]) AND ("牙髓炎"[常用字段:智能] OR "牙髓炎症"[常用字段:智能]) AND ("酮咯酸"[常用字段:智能] OR "酮咯酸氨丁三醇"[常用字段:智能])

Number of detected records：17

1. **WanFang：**

检索表达式（中英文扩展&主题词扩展）：主题:(酮咯酸 or 酮咯酸氨丁三醇) and 主题:(牙髓炎 or 牙髓炎症) and 主题:(疼痛 or 痛证 or 牙痛 or 牙疼痛)

Number of detected records：92

1. **CNKI:**

（主题：酮咯酸 + 酮咯酸氨丁三醇）AND（主题：牙髓炎 + 牙髓炎症）AND（主题：疼痛+痛证+牙痛+牙疼痛）

Number of detected records：0
